# SIRT1 is a positive regulator of *in vivo* bone mass and a therapeutic target for osteoporosis

**DOI:** 10.1371/journal.pone.0185236

**Published:** 2017-09-22

**Authors:** Kayvan Zainabadi, Cassie J. Liu, Alison L. M. Caldwell, Leonard Guarente

**Affiliations:** Glenn Center for the Science of Aging, Department of Biology, Koch Institute, MIT, Cambridge, Massachusetts, United States of America; Kyungpook National University School of Medicine, REPUBLIC OF KOREA

## Abstract

Overexpression or pharmacological activation of SIRT1 has been shown to extend the lifespan of mice and protect against aging-related diseases. Here we show that pharmacological activation of SIRT1 protects in two models of osteoporosis. Ovariectomized female mice and aged male mice, models for post-menopausal and aging-related osteoporosis, respectively, show significant improvements in bone mass upon treatment with SIRT1 agonist, SRT1720. Further, we find that calorie restriction (CR) results in a two-fold upregulation of *sirt1* mRNA expression in bone tissue that is associated with increased bone mass in CR mice. Reciprocally, SIRT1 whole-body knockout (KO) mice, as well as osteoblast and osteoclast specific KOs, show a low bone mass phenotype; though double knockout mice (containing SIRT1 deleted in both osteoblasts *and* osteoclasts) do not show a more severe phenotype. Altogether, these findings provide strong evidence that SIRT1 is a positive regulator of bone mass and a promising target for the development of novel therapeutics for osteoporosis.

## Introduction

The SIR2 family of proteins (named the Sirtuins) are NAD^+^ dependent enzymes that are evolutionarily conserved from yeast to humans [1]. Yeast SIR2 utilizes NAD^+^ as a co-substrate to catalyze deacetylation of histone proteins, thereby linking metabolism with gene silencing [2]. Importantly, increased dosage and/or pharmacological activation of SIR2 orthologues have been shown to extend the lifespan of model organisms, including mice [3–10]. The mammalian genome contains seven Sirtuins, of which SIRT1 shares the greatest sequence similarity with yeast SIR2. Activation of SIRT1 in mice is associated with a delay in the onset of many aging-related diseases, including osteoporosis [10–19].

Another regimen that extends lifespan in a wide variety of organisms is calorie restriction (CR). Mice fed a diet 30–40% below *ad libitum* (AL) levels live longer and are more resistant to the development of a number of aging-related diseases [20]. Evidence has emerged that SIRT1 may act as an important mediator of the beneficial effects of CR [21]. For instance, SIRT1 knockout mice fail to display a number of phenotypes associated with CR, including increased activity, higher respiration, and lifespan extension [22–24]. Importantly, CR has been shown to stimulate the expression and activity of SIRT1 in various mammalian tissues, suggesting activation of SIRT1 may mimic the effects of CR [25–26]. Consistent with this, mice treated with SIRT1 agonists show a transcriptional profile that closely mirrors that of CR [16–17]. While CR has been shown to protect against a number of aging-related diseases, its role in osteoporosis has been far less conclusive [27–30].

Osteoporosis is a classic aging-related disease that occurs due to an imbalance in bone remodeling. This can arise either through hyperactivation of osteoclasts (the hematopoietic cells that break down bone) or hypoactivation of osteoblasts (the mesenchymal cells that form bone), resulting in post-menopausal (or Type 1) or aging-related (or Type 2) osteoporosis, respectively. There are currently no effective treatments for aging-related osteoporosis [31].

To better define the role of SIRT1 in bone remodeling, we have taken a systematic *in vivo* approach. Using both whole-body and tissue-specific knockouts, we show that deletion of SIRT1 invariably results in an osteoporotic phenotype. Reciprocally, short-term pharmacological activation of SIRT1 in two models of osteoporosis leads to significant increases in bone mass. Interestingly, calorie restriction also results in improved bone mass that is associated with increased expression of SIRT1 in bone tissue. Our data solidify SIRT1 as an important regulator of bone mass and promising target for the treatment of osteoporosis.

## Materials and methods

### Animal experimentation

All mice were cared for in accordance with the MIT Committee on Animal Care (MITCAC), which approved this study. Mice were housed under controlled temperature (25 ± 1°C) and lighting conditions, and fed standard chow *ad libitum* unless otherwise stated.

Sirt1^flox^*/*^flox^ mice and osteoclast Cre mice (Lysozyme M promoter) were obtained from Jackson Laboratory; osteoblast specific Cre mice (2.3kb Collagen type 1 promoter) were obtained from the Mutant Mouse Regional Resource Center (MMRRC). Whole-body knockouts were in a CD1 and 129/Sv mixed background (due to lethality in an inbred background), osteoblast knockouts were in a mixed C57BL/6 × BALB/c background, and osteoclast knockouts and Sirt1^flox^*/*^flox^ were in a C57BL/6 background. Female mice were used for all experiments unless otherwise stated.

Calorie restricted (CR) C57BL/6J male mice that had been on a 60% *ad libitum* diet for 4 months starting at 4 months of age were obtained from the National Institutes of Aging (NIA). Once received, mice were kept on CR for an additional 2 weeks in order to re-equilibrate mice to the new surroundings.

For SRT1720 feeding experiments, 12 month old male C57BL/6J mice were fed 100mg/kg/day SRT1720 (SirTris) [32] or vehicle control in their chow for 5 months. 3 month old ovariectomized or sham treated C57BL/6J female mice were obtained from Jackson Laboratories and similarly fed for 1 month with SRT1720. Mice were euthanized with carbon dioxide asphyxiation as approved by MITCAC and bones fixed in 10% formalin overnight at 4°C, and then stored in 70% ethanol.

### Microcomputed tomography (μCT) analysis

Dissected bones were analyzed by μCT using a Skyscan 1172 instrument (60kV, 150μA, using a 0.5-mm aluminum filter) at a resolution of 5μm. Measurements were taken from 200 slices immediately distal of the primary spongiosa and analyzed for alterations in trabecular bone parameters. Resulting images were reconstructed using the Skyscan NRecon program and analyzed using Skyscan CTAN software. Analyses were performed typically for the femur or tibia, except for double knockout mice (DKO) for which analysis was only performed for the spine.

### Quantitative reverse-transcriptase polymerase chain reaction (qRT-PCR)

Total RNA was extracted from cells or tissues using Trizol (Invitrogen) and the RNeasy MinElute Cleanup kit (Qiagen). 1μg of RNA was used for cDNA synthesis using random hexamers with the SuperScript III reverse transcriptase kit (Invitrogen). cDNA was then subjected to quantitative PCR analysis in the presence of iQ-SYBR green (Bio-Rad). Relative mRNA abundance was obtained by normalization to ribosomal *rpl19* levels.

For RNA extraction from bone tissue, calvaria (top of the skull) of mice was dissected out, washed with cold PBS to remove non-osseous tissue, and then flash-frozen in liquid nitrogen. Calvaria was then minced in Trizol, and homogenized using a Tissue Tearor homogenizer (VWR) in the fume hood. Lysates were then spun down at 15,000g for 10 minutes, with the resulting supernatant used for RNA isolation using the RNeasy MinElute Cleanup kit (Qiagen).

### Statistical analysis

Statistical analysis was performed using an unpaired Student’s t test, with significant differences indicated by single asterisk (*) when p < 0.05, double asterisk (**) when p < 0.01, and triple asterisk (***) when p < .005. All data is presented ± standard error of the mean (SEM).

## Results

### SIRT1 knockout mice display a low bone mass phenotype

**Fig 1:**
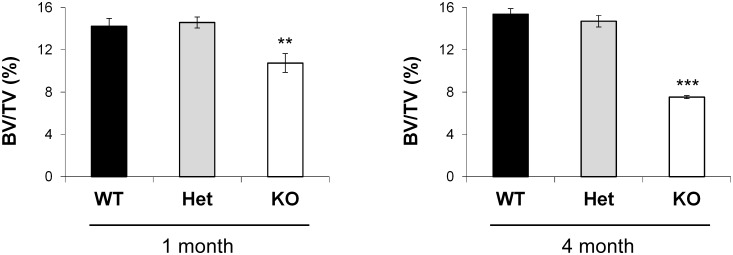
Deletion of SIRT1 leads to a low bone mass phenotype. SIRT1 whole-body knockout mice show decreased bone volume/total volume (BV/TV) at both 1 month and 4 months of age as assessed by microcomputed tomography (μCT). (n ≥ 4 for each group; ** p < .01; *** p < .005).

To comprehensively address the role of SIRT1 in bone remodeling, we first analyzed whole-body SIRT1 knockout mice using microcomputed tomography (μCT). We found that SIRT1 knockout mice show marked reductions in bone mass at both 1 month and 4 months of age, with **t**he phenotype appearing to worsen with age (Fig 1). Heterozygous knockout mice did not show any differences in bone mass at either age (Fig 1).

**Fig 2:**
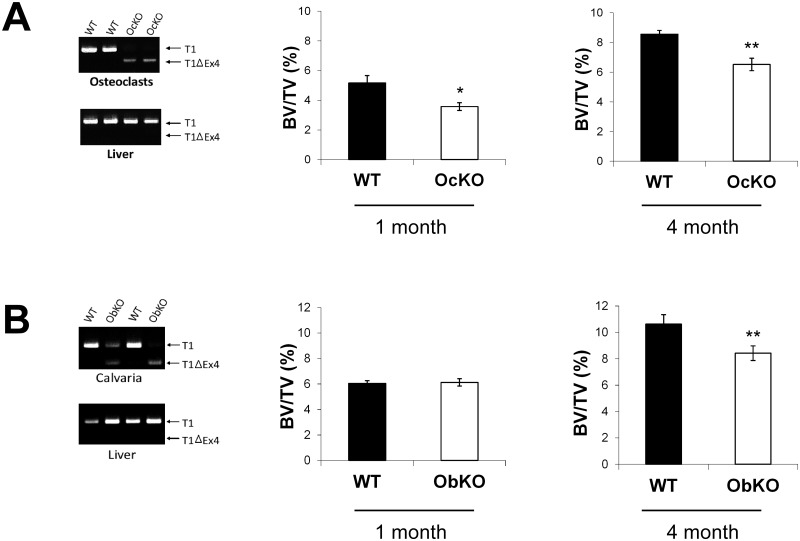
Deletion of SIRT1 in osteoclasts or osteoblasts results in a low bone mass phenotype. (A) Osteoclast tissue specific SIRT1 knockout mice (OcKO) show excision of SIRT1 in osteoclasts, but not liver, as indicated by a smaller PCR product obtained with primers flanking SIRT1 catalytic exon 4 (T1Δ4). OcKOs show decreased bone mass at both 1 month and 4 months of age. (B) Osteoblast tissue specific SIRT1 knockout mice (ObKO) mice show excision of SIRT1 in calvaria, but not liver. ObKOs show decreased bone mass only at 4 months of age. (n ≥ 5 for each group; * p < .05; ** p < .01).

To determine whether this phenotype is due to a cell-autonomous effect, we next generated tissue specific SIRT1 knockout mice. To obtain osteoblast specific SirT1 knockouts (ObKO), we crossed SirT1^flox^*/*^flox^ animals with mice expressing Cre under the 2.3kb Collagen type 1 promoter [33]. This well-characterized promoter is specific to osteoblasts that have exited the cell cycle and are actively undergoing differentiation [34]. To generate osteoclast specific knockouts (OcKO) we used the previously described Lysozyme-M Cre promoter which specifically targets the monocyte precursors that give rise to osteoclasts [35]. In each case, expression of Cre leads to excision of the catalytic domain of SIRT1 in osteoblasts or osteoclasts, respectively, but not in other tissue types such as the liver (Fig 2A and 2B).

Unlike SIRT1 whole-body knockouts, ObKO and OcKO mice were obtained at expected Mendelian ratios and were indistinguishable from wildtype littermates, though we did note a modest but statistically significant increase in blood glucose levels for ObKOs (but not OcKOs) (S1 Fig). μCT analysis showed that OcKO mice displayed reduced bone mass at both 1 month and 4 months of age (Fig 2A). In contrast, ObKOs showed an osteoporotic phenotype only at 4 months of age (Fig 2B).

**Fig 3:**
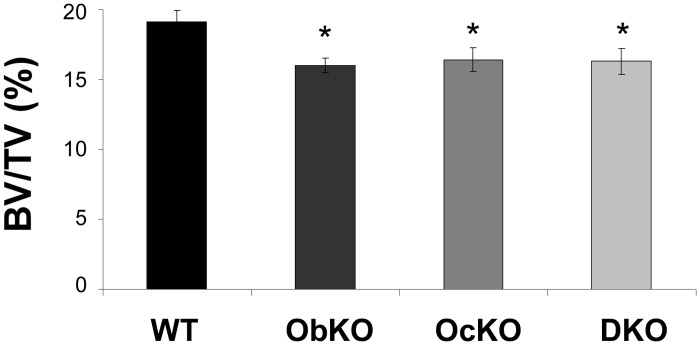
Deletion of SIRT1 in both osteoblasts and osteoclasts does not lead to a more severe phenotype. Adult double knockout mice (DKO) containing SIRT1 deleted in both osteoblasts and osteoclasts show similar bone deficits as compared to individual osteoblast (ObKO) or osteoclasts (OcKO) knockout mice. (n ≥ 6 for each group; * p < .05).

To determine whether deletion of SIRT1 in both cell types would result in an even more severe phenotype, we crossed ObKO and OcKO mice in order to obtain double knockouts (DKO). Somewhat surprisingly, adult DKOs did not show an even more pronounced phenotype as each individual knockout (Fig 3). Altogether, these data indicate that SIRT1 positively regulates bone mass, at least in part in a cell-autonomous manner, consistent with previous studies [36–39].

### Pharmacological activation of SIRT1 protects in two models of osteoporosis

**Fig 4:**
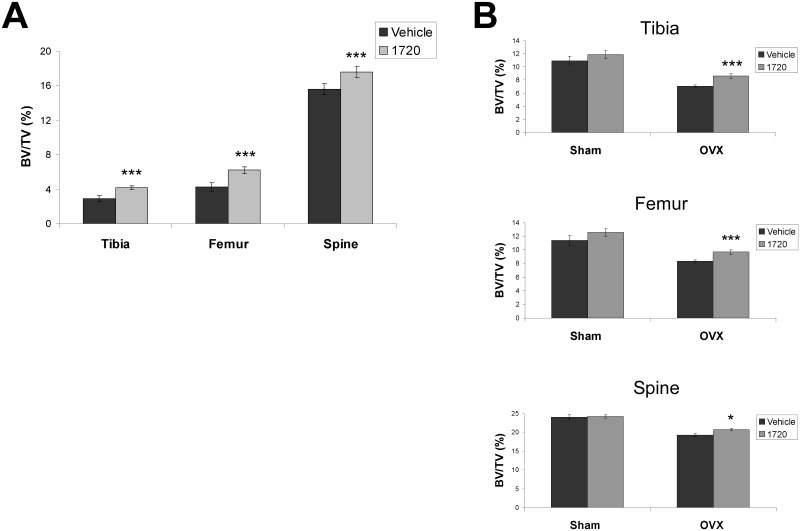
SIRT1 agonist, SRT1720, increases bone mass in aged male mice and ovariectomized female mice. (A) 12 month old male mice treated for 5 months with 100mg/kg/day of SIRT1 agonist, SRT1720, show increased bone mass compared to mice treated with vehicle control (final age 17 months). (B) Ovariectomized (OVX) female mice treated with SRT1720 for 1 month show a modest but significant increase in bone mass compared to vehicle treated controls (final age 4 months). (n ≥ 9 for each group; * p < .05; ** p < .01; *** p < .005).

Given that loss of SIRT1 resulted in decreased bone mass, we next explored whether activation of SIRT1 could increase bone mass, particularly in models of osteoporosis. To determine the effects of SIRT1 activation on age-related osteoporosis, we treated 12 month old male C57BL/6 mice for 5 months with SIRT1 agonist SRT1720 (100mg/kg/day) [32]. We found that mice fed SRT1720 showed significant improvements in bone mass as compared to vehicle control animals (Fig 4A).

We next examined the effect of SIRT1 activation in a model of post-menopausal osteoporosis. This is a more acute model of osteoporosis as ovariectomized (OVX) mice lose 20–30% of their bone mass in just one month (Fig 4B). OVX mice were treated with 100mg/kg/day SRT1720 for one month immediately after their surgery. In contrast to animals treated with vehicle control, mice treated with SRT1720 showed a significant amelioration of the OVX-induced bone loss (Fig 4B). The effect was a partial rescue and a higher dose of SRT1720 (200mg/kg/day) did not improve this rescue any further.

### Calorie restriction leads to increased bone mass associated with upregulation of SIRT1 expression

**Fig 5:**
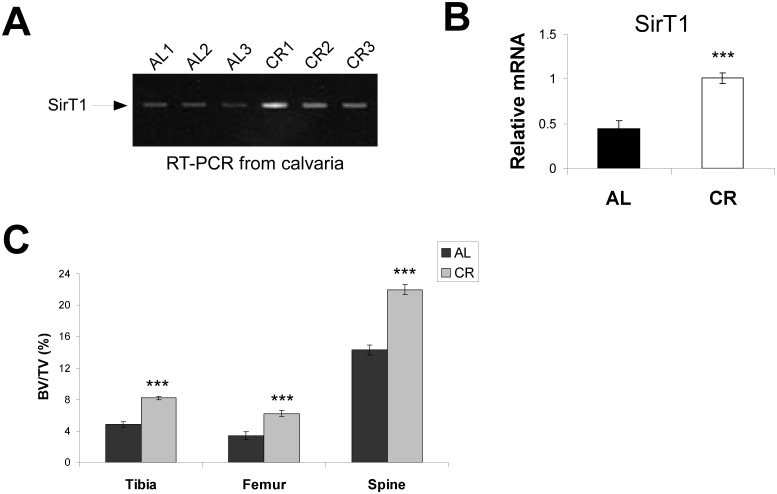
Calorie restriction (CR) leads to upregulation of SIRT1 expression and increased bone mass. (A) Calorie restricted (CR) mice show increased expression of SIRT1 in whole calvaria as assessed by reverse transcriptase polymerase chain reaction (RT-PCR). (B) Quantitative RT-PCR (qRT-PCR) confirms a two-fold increase in expression of SIRT1 (after normalization to RPL19) in the calvaria of CR mice. (C) 8 month old CR mice (placed on a CR diet for 4 months at 4 months of age) show marked increases in bone mass in all bones examined as compared to *ad libitum* (AL) fed controls. (n ≥ 4 for each group; *** p < .005).

Calorie restriction (CR) has been shown to forestall many diseases of aging, though its effects on osteoporosis have been more ambiguous [27–30]. In an attempt to resolve this, we obtained CR mice from the central colony maintained at the National Institutes of Aging (NIA). The mice (C57BL/6) used in this study were 8 months old and had been placed on a CR diet consisting of 60% *ad libitum* (AL) levels for 4 months (starting at 4 months of age). As CR has previously been shown to induce SIRT1 expression in certain tissues [25–26], we first analyzed the expression of SIRT1 in the calvaria (skullcap) of CR mice. We found a two-fold upregulation in SIRT1 mRNA expression in the calvaria of CR mice as compared to AL animals (Fig 5A and 5B). To determine the effects of CR on bone mass, we next performed μCT and found that CR mice showed a marked increase in bone mass for all bones examined (Fig 5C).

### SIRT1 expression does not change with age in bone

**Fig 6:**
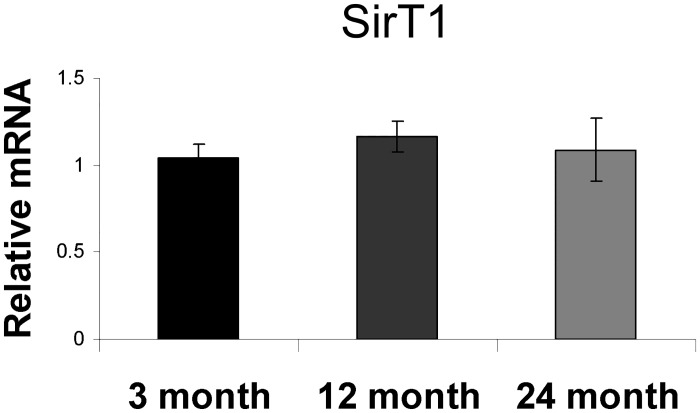
SIRT1 expression in bone tissue does not change with age. Expression of SIRT1 mRNA in the calvaria of mice remains constant with age. (n = 4 for each group).

SIRT1 expression has been reported to decline in certain tissues with aging [39–41]. To determine if a similar mechanism was at play in the skeleton, we next analyzed the expression of SIRT1 in the calvaria of young (3 month), old (12 month), and very old (24 month) C57BL/6 mice obtained from the NIA. In contrast to a previous report [39], we were unable to find any differences in SIRT1 mRNA expression in the bones of young versus old mice (Fig 6).

## Discussion

Activation of SIRT1 has previously been shown to increase the healthspan and delay the onset of many aging-related diseases in mice [7–19]. Here we examined the role of SIRT1 in osteoporosis, a classic aging-related disease, by taking a comprehensive *in vivo* approach. One shortcoming of this approach, however, is that we were unable to perform histology and thus bone histomorphometry data such as *in vivo* osteoblast and osteoclast counts is lacking. Nonetheless, we did find that deletion of SIRT1 invariably resulted in a low bone mass phenotype, consistent with previous studies [36–39]. Since deletion of SIRT1 in osteoblasts and osteoclasts also resulted in low bone mass, this indicates that SIRT1 regulates bone remodeling at least in part in a cell-autonomous fashion. While whole-body knockout mice did display a more severe phenotype than any of the tissue specific knockouts, direct comparisons between the two are difficult owing to the different backgrounds of the mice (deletion of SIRT1 in an inbred background is lethal). It should, however, be noted that SIRT1 has previously been shown to affect non-cell autonomous pathways such as the somatotropic and endocrine axis [42–45] which would be expected to impinge on bone mass. In fact, SIRT1 overexpression in the brain was recently shown to be sufficient to extend the lifespan of mice, highlighting the powerful non-cell autonomous role SIRT1 plays in organismal biology [9]. It will therefore be interesting to determine the precise cell autonomous and non-autonomous role SIRT1 plays in bone remodeling, and whether any cross-talk (particularly in response to nutritional state) exists between the two. The mouse models developed in this study should be helpful in addressing these questions for future studies.

Unlike its widely accepted role in delaying the onset of aging-related diseases, the role of CR on bone mass has been somewhat murky. Some studies have shown a positive effect, while others have found either a negative or no effect at all [27–30]. This is likely due to the previous use of varying CR protocols, animal models, and strain backgrounds. Here, we have used mice from a central repository at the National Institutes of Aging (NIA) so as to use a standard model easily accessible by the scientific community. We show that mice in which CR was initiated during adulthood (at 4 months of age), and maintained for at least 4 months, show a marked increase in bone mass. A similar effect was observed for mice maintained for a longer 8 month period as well. This increased bone mass was associated with a two-fold induction of SIRT1 expression in the bone tissue of CR mice, similar to that observed for other tissues with CR [25–26]. At first glance, this appears as a rather peculiar finding—mice on a CR diet generally enter a catabolic state, resulting in loss of significant body weight. However, it is important to note that not all tissues shrink during CR: for example while the liver and white adipose tissue reduce in mass, the brain and heart do not [20]. These results, therefore, indicate that CR is more likely inducing a regulated response redirecting resources to different tissues and organs in order to meet the needs of the new low caloric diet. Behavior changes such as increased physical activity, presumably a foraging response, associated with CR are an example of this, and we believe the observed changes in bone mass are yet another example (and perhaps the two are related).

Most excitingly, pharmacological activation of SIRT1 resulted in significant increases in bone mass in two independent models of osteoporosis. Treating 12 month-old mice with SRT1720 for 5 months resulted in an almost 30% increase in femoral bone mass; a site of frequent fractures in osteoporotic humans. While the differences in bone mass in ovariectomized (OVX) mice were not as dramatic, it should be noted that these mice were much younger (3 months) and were treated for only 1 month. Nonetheless, even with this short treatment, OVX mice showed a highly significant increase in bone mass as compared to vehicle treated animals. As the bone loss from ovariectomy is almost entirely due to increased osteoclast activity, it is reasonable to infer that the partial rescue is due to inhibition of osteoclast activity by activation of SIRT1. Consistent with this model, sham treated mice did not show any improvements in bone mass, indicating that SIRT1 is specifically acting to protect from OVX-induced bone loss. These findings are in agreement with previous studies showing SIRT1 acts as a negative regulator of both osteoclast activity and osteoclastogenesis [39, 46–47]. Encouragingly, a recent study using a more potent SIRT1 agonist resulted in an even more dramatic rescue in a similar OVX model of osteoporosis [48].

Unlike a previous report [36], we did not find SIRT1 expression to decrease with age in bone. We attribute this discrepancy to the following: 1) We used calvaria for our qRT-PCR analysis, whereas the aforementioned study used whole long bones where the majority of RNA comes from hematopoietic and mesenchymal precursor cells in the marrow; 2) We used ribosomal gene *rpl19* for normalization, whereas the Edwards *et al* normalized with the metabolically relevant *gapdh* gene, the expression of which has previously been shown to change with age, nutritional status, and mechanical strain on bone [49–51]. In contrast, expression of *rpl19* has been shown to be more constant under a variety of conditions [52–53], including during normal aging [54]. Regardless, the effect of aging on SIRT1 protein levels and activity in bone tissue is still an open question.

Overall, the data presented here support the model that SIRT1 acts as a positive regulator of *in vivo* bone mass. These findings add to our previous work showing that SIRT1 promotes osteoblast commitment and differentiation by positively regulating β-catenin and RUNX2, respectively [36, 55]. In fact, RUNX2 targets have previously been shown to be upregulated in bone tissue upon SIRT1 activation, consistent with an anabolic role for SIRT1 [55]. The current demonstration of the *in vivo* efficacy of the SIRT1 agonist SRT1720 on bone mass adds weight to the idea that this Sirtuin is a strong pharmacological target for treating both age-related and post-menopausal osteoporosis. Encouragingly, a recent human trial showed that treatment with resveratrol, another SIRT1 activator, led to a statistically significant increase in bone mass in elderly obese men [56]. Therefore new SIRT1 agonists, one of which is already in clinical trials [57–59], may pave the way towards novel therapeutics for osteoporosis.

## Supporting information

S1 Fig Deletion of SIRT1 in osteoblasts leads to increased blood glucose levels. Osteoblast specific SIRT1 knockouts (ObKO) show higher blood glucose levels as compared to wildtype littermate controls under fed, but not fasted, conditions. Osteoclast specific knockouts (OcKO) do not show a difference under either condition. (n ≥ 9 for each group; * p < .05).
(TIF)
