# Supplementary figures and images for: SIRT1 is a positive regulator of in vivo bone mass and a therapeutic target for osteoporosis
Source: PLoS One. 2017 Sep 22;12(9):e0185236. doi: 10.1371/journal.pone.0185236 (PMC5609767; doi:10.1371/journal.pone.0185236)

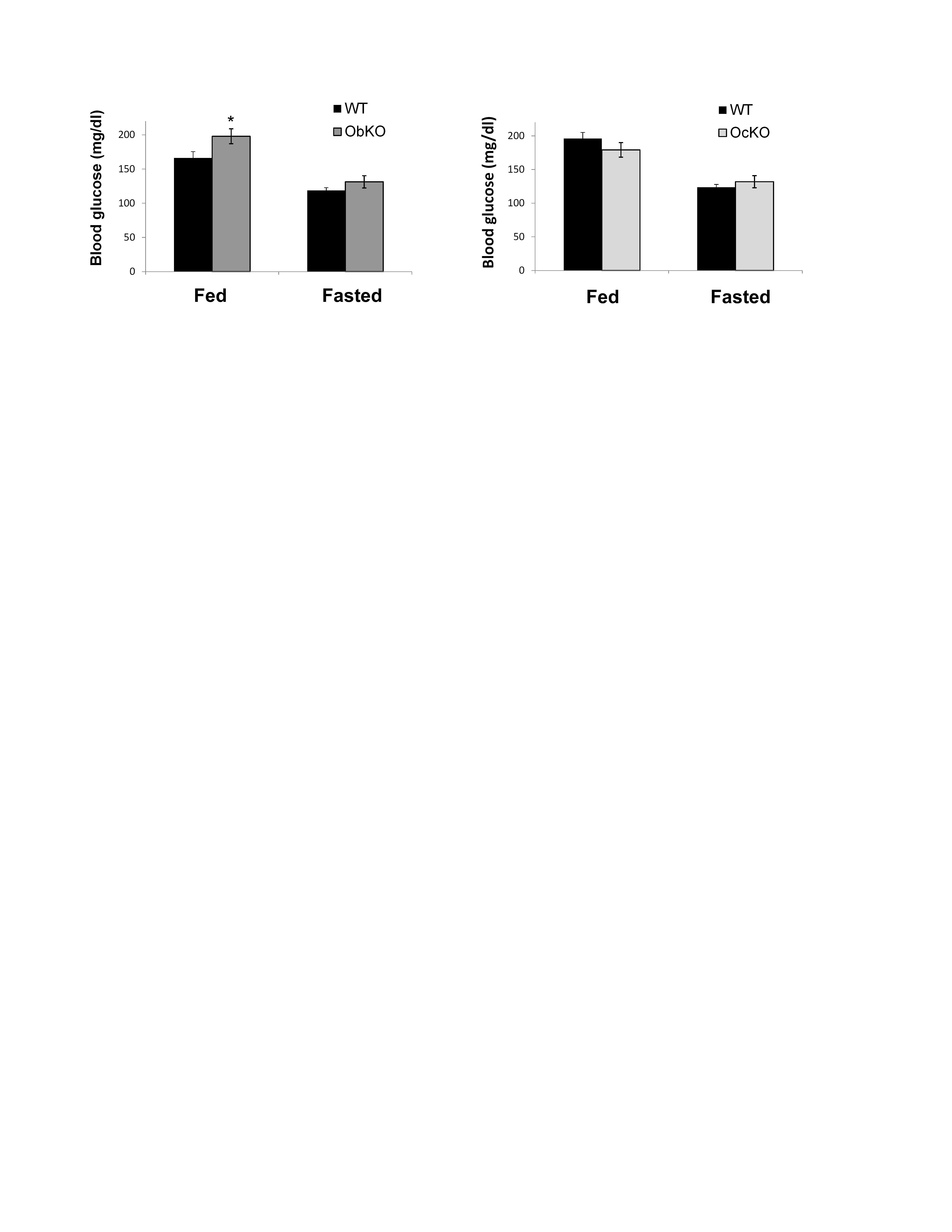

Supplement: S1 Fig — Osteoblast specific SIRT1 knockouts (ObKO) show higher blood glucose levels as compared to wildtype littermate controls under fed, but not fasted, conditions. Osteoclast specific knockouts (OcKO) do not show a difference under either condition. (n ≥ 9 for each group; * p < .05). (TIF) [file pone.0185236.s001.tif]
